# *Helicobacter pylori* Induces IL-33 Production and Recruits ST-2 to Lipid Rafts to Exacerbate Inflammation

**DOI:** 10.3390/cells8101290

**Published:** 2019-10-21

**Authors:** Chia-Jung Kuo, Chun-Ya Chen, Horng-Ren Lo, Chun-Lung Feng, Hui-Yu Wu, Mei-Zi Huang, Tung-Nan Liao, Yu-An Chen, Chih-Ho Lai

**Affiliations:** 1 Department of Gastroenterology and Hepatology, Chang Gung Memorial Hospital at Linkou, Taoyuan 33305, Taiwan; m7011@cgmh.org.tw; 2 Department of Microbiology and Immunology, Graduate Institute of Biomedical Sciences, College of Medicine, Chang Gung University, Taoyuan 33302, Taiwan; magicgirl0203@gmail.com (C.-Y.C.); winney0614@gmail.com (H.-Y.W.); s05060714@gmail.com (M.-Z.H.); yu-an.chen@utsouthwestern.edu (Y.-A.C.); 3 Department of Laboratory Medicine, Taichung Veterans General Hospital Chiayi Branch, Chiayi 60090, Taiwan; 4 Department of Medical Laboratory Science and Biotechnology, Fooyin University, Kaohsiung 83102, Taiwan; MT096@fy.edu.tw; 5 Division of Gastroenterology and Hepatology, Department of Internal Medicine, China Medical University Hsinchu Hospital, Hsinchu 30272, Taiwan; fjld6604@yahoo.com.tw; 6 Department of Microbiology, School of Medicine, China Medical University and Hospital, Taichung 40402, Taiwan; 7 Department of Medical Laboratory Science and Biotechnology, Chung Hwa University of Medical Technology, Tainan 71703, Taiwan; tn20030303@yahoo.com.tw; 8 Department of Urology, University of Texas Southwestern Medical Center, Dallas, TX 75390, USA; 9 Department of Nursing, Asia University, Taichung 41354, Taiwan; 10 Department of Pediatrics, Molecular Infectious Disease Research Center, Chang Gung Memorial Hospital at Linkou, Taoyuan 33305, Taiwan

**Keywords:** *Helicobacter pylori*, IL-33, ST-2, inflammation, lipid rafts

## Abstract

*Helicobacter pylori* colonizes human gastric epithelial cells and contributes to the development of several gastrointestinal disorders. Interleukin (IL)-33 is involved in various immune responses, with reported proinflammatory and anti-inflammatory effects, which may be associated with colitis and colitis-associated cancer. IL-33 induces the inflammatory cascade through its receptor, suppression of tumorigenicity-2 (ST-2). Binding of IL-33 to membrane-bound ST-2 (mST-2) recruits the IL-1 receptor accessory protein (IL-1RAcP) and activates intracellular signaling pathways. However, whether IL-33/ST-2 is triggered by *H. pylori* infection and whether this interaction occurs in lipid rafts remain unclear. Our study showed that both IL-33 and ST-2 expression levels were significantly elevated in *H. pylori*-infected cells. Confocal microscopy showed that ST-2 mobilized into the membrane lipid rafts during infection. Depletion of membrane cholesterol dampened *H. pylori*-induced IL-33 and IL-8 production. Furthermore, in vivo studies revealed IL-33/ST-2 upregulation, and severe leukocyte infiltration was observed in gastric tissues infected with *H. pylori*. Together, these results demonstrate that ST-2 recruitment into the lipid rafts serves as a platform for IL-33-dependent *H. pylori* infection, which aggravates inflammation in the stomach.

## 1. Introduction

*Helicobacter pylori* is a Gram-negative, spiral-shaped, microaerophilic bacteria that colonizes the human stomach and infects more than 50% of the human population worldwide [1]. Patients infected with *H. pylori* typically present with gastrointestinal-associated disorders, such as chronic gastritis, peptic ulcer, and gastric adenocarcinoma [2]. *H. pylori* induces gastric inflammation through the activation of the nuclear factor-κB (NF-κB) signaling pathway in gastric epithelial cells, followed by the secretion of proinflammatory cytokines, such as IL-1, IL-6, IL-8, and tumor necrosis factor (TNF)-α [3].

IL-33 is a member of the IL-1 family that is produced during tissue damage and functions as an alarmin [4]. Binding of IL-33 to membrane-bound suppression of tumorigenicity-2 (mST-2) recruits the IL-1 receptor accessory protein (IL-1RAcP) and subsequently activates the NF-κB and mitogen-activated protein kinase (MAPK) signaling pathways in Th2 and mast cells [5]. Inflammatory proteases of microbe-infected cells cleave the full-length IL-33 into a processed form of IL-33, which effectively enhances immune cell activation and release of proinflammatory cytokines [6]. Additionally, IL-33-deficient mice have been reported to be highly associated with colitis and colitis-associated cancer, indicating that IL-33 has a protective effect in intestinal immunity [7]. In contrast, IL-33 plays a role in anti-inflammatory processes as a potent activator of M2 macrophages and in regulatory T-cell (Treg) differentiation [8]. Therefore, IL-33 possesses a dual role that orchestrates both proinflammatory and anti-inflammatory effects during microbial infections.

*H. pylori* infection upregulated mucosal IL-33 mRNA expression in patients with gastritis, indicating that IL-33 exacerbates the inflammatory response in the gastric mucosa [9]. Furthermore, NOD1 signaling was implicated in IL-33 production by *H. pylori*-infected gastric epithelial cells [10]. Moreover, IL-33 also induced TNF-α production by mast cells, which facilitated *H. pylori* colonization and worsened gastritis [11]. Together, these indicate that IL-33 can intrinsically manipulate the immune system in response to *H. pylori* infection.

Lipid rafts are unique membrane microdomains containing high concentrations of cholesterol, gangliosides, sphingomyelin, and copious amounts of proteins that respond to microbial infections [12,13]. Several *H. pylori* virulence factors, such as cytotoxin-associated gene A (CagA) and vacuolating cytotoxin A (VacA), which induce pathogenesis [14], are closely associated with membrane lipid rafts [15,16,17,18,19]. Disruption of lipid rafts by cholesterol disruptors/usurpers abolishes *H. pylori* virulence and alleviates its related morbidity [20,21,22]. However, the involvement of membrane rafts in IL-33/ST-2-dependent *H. pylori*-induced inflammation remains unclear. This study explored the role of IL-33 and its receptor suppression of tumorigenicity-2 (ST-2) in *H. pylori* infection in gastric epithelial cells. Furthermore, we investigated whether *H. pylori* exploits lipid rafts to induce IL-33/ST-2 signaling for facilitating inflammation in gastric epithelial cells.

## 2. Materials and Methods

### 2.1. H. pylori and Cell Culture

*H. pylori* 26695 (ATCC 700392 with CagA^+^/VacA^+^) was used as the reference strain, which has been characterized as described previously [23]. The bacteria were routinely cultured on 10% sheep blood agar plates in a microaerophilic environment (85% N_2_, 10% CO_2_, 5% O_2_) for 24 to 36 h at 37 °C to achieve optimum microbial activity and then subjected to the cell infection experiments [19]. To perform the study of *H. pylori*-infected gastric epithelial cells, human gastric epithelial cells (AGS cells, ATCC CRL 1739) were cultured in F12 medium (Hyclone, Logan, UT, USA). SCM-1 cells and TSGH9201 cells (BCRC 60146) were cultured in RPMI1640 medium (Hyclone) [21]. All culture medium was supplemented with 10% complement-inactivated fetal bovine serum (Hyclone). Antibiotics were not added to the cell culture medium in *H. pylori*-infected assay.

### 2.2. Western Blot Analysis

To investigate the protein expression levels of IL-33, ST-2, and IL-1RAcP in the *H. pylori*-infected human gastric epithelial cells, Western blot analysis was employed. AGS cells (4 × 10^5^) were seeded in 6-well plates and infected with *H. pylori* at the assigned multiplication of infection (multiplication of infection ) for the indicated time. *H. pylori*-infected cells were lyzed by RIPA (150 mM NaCl, 50 mM Tris base pH7.4, and 1 mM EDTA, 1% NP-40, 0.25 mM deoxycholate). The samples (50 µg/ sample) were resolved by 10% SDS-PAGE and transferred onto polyvinylidene difluoride membranes (Millipore, Billerica, MA, USA). The membranes were blocked by 5% defatted milk with TBS-T (TBS buffer containing 0.1% Tween 20) at room temperature for 90 minutes. The membranes were incubated with mouse anti-IL-33 antibody (Proteintech, Chicago, IL, USA) to recognized full-length IL-33 (36 KDa) and processed form of IL-33 (18 KDa), rabbit anti-ST-2 antibody (MyBioSource, San Diego, CA, USA), and mouse anti-IL-1RAcP antibody (Santa Cruz Biotechnology, Santa Cruz, CA, USA) at 4 °C overnight. The blots were washed and then incubated with a horseradish peroxidase-conjugated secondary antibody (Millipore). The proteins of interests were detected using ECL Western blotting detection reagents (GE Healthcare, Piscataway, NJ, USA) and visualized the signals using an Azure c400 system and AzureSpot Analysis Software (Azure Biosystems, Dublin, CA, USA) following the manufacturer’s instructions [24].

### 2.3. Quantitative Real-Time Reverse Transcription-PCR

To explore the mRNA levels of IL-33/ST-2 in the *H. pylori*-infected gastric epithelial cells, quantitative real-time PCR (qRT-PCR) analysis was used in this study. IL-33 and ST-2 mRNA levels were analyzed by qRT-PCR using SYBR Green I Master Mix and a model 7900 Sequence Detector System. The oligonucleotide primers corresponded to human IL-33 (forward, 5’-GGAAGA ACACAGCAAGCAAAGCCT-3’ and reverse, 5’-TAAGGCCAGAGCGGAGCTTCATAA-3’) and human mST-2 (forward, 5’- ACAAAGTGCTCTACACGACTG-3’ and reverse, 5’- TGTTCTGGA TTGAGGCCAC-3,) and glyceraldehyde-3-phosphate dehydrogenase (GAPDH) (forward, 5′-CCCCCAATGTATCCGTTGTG-3’ and reverse, 5’-TAGCCCAGGATGCCCTTTAGT-3’) [25]. The program was pre-incubated at 50 °C for 2 min and 95°C for 10 min; PCR was performed with 40 cycles of 95 °C for 10 s and 60 °C for 1 min.

### 2.4. Immunofluorescence Labeling and Confocal Microscopic Analysis

AGS cells (2 × 10^5^) were seeded on coverslips in 6-well plates followed by *H. pylori* infection at an MOI of 100 for 9 h. The infected cells were washed with 1 × PBS and fixed with 1% paraformaldehyde at room temperature for 1 h and then permeabilized with 0.1% Triton X-100 for 10 min. The fixed cells were blocked with 3% cosmic calf serum (Hyclone) for 1 h then incubated with antibodies specific to IL-33 (Proteintech), ST-2 (Proteintech), and IL-1RAcP (Santa Cruz Biotechnology), respectively, for 1 h 30 min. The cells were then probed with Alexa Fluor 568-conjugated goat anti-mouse IgM, Alexa Fluor 488-conjugated goat anti-rabbit IgG, or Alexa Fluor 488-conjugated goat anti-mouse IgG, respectively, for 1 h. The stained cells were analyzed using confocal microscopy (LSM 780; Carl Zeiss, Göttingen, Germany) with a 100× objective (oil immersion; numerical aperture, 1.3) [26]. All image analyses and processing were performed with the ZEN-blue edition software (Carl Zeiss).

### 2.5. Fractionation of Cytoplasmic and Nuclear Proteins

AGS cells (4 × 10^5^) were seeded in 6-well plates and infected with *H. pylori* at an MOI of 100 for 9 h. Cytoplasmic and nuclear proteins were obtained with the NE-PER nuclear and cytoplasmic extraction kit (Thermo Scientific, Barrington, IL, USA) according to the manufacturer’s protocol. IL-33 levels in either cytoplasmic or nuclear fraction were measured by ELISA with the human IL-33 DuoSet kit (R&D Systems, Minneapolis, MN, USA).

### 2.6. Determination of Cytokine Production

The cytokine levels of IL-8 and IL-33 were determined by enzyme-linked immunosorbent assay (ELISA) as described previously [27]. Briefly, AGS cells were treated with cholesterol depletion/usurpation agents, including: methyl-β-cyclodextrin (MβCD, 5.0 mM), simvastatin (10 µM), and nystatin (50 µg/mL) [16], respectively, followed by infection with *H. pylori* (MOI = 100) for 9 h. The concentration of each cytokine was determined using a sandwich ELISA kit (R&D Systems).

### 2.7. Animal Study

Male BALB/c mice aged 6-week-old were purchased from the National Laboratory Animal Center (Taipei, Taiwan). The animal study was conducted in accordance with the Laboratory Animal Center of Chang Gung University under a protocol approved by the Institutional Animal Care Use Committee (IACUC Approval No.: CGU16-004). The experiment was performed from 1 June 2016 to 31 December 2018, in accordance with the institutional guidelines. The experiments with animals were performed in the Laboratory Animal Center of Chang Gung University. Mice were divided into two groups: PBS control treatment (n = 6) and *H. pylori* infection (n = 4). Mice were administered *H. pylori* (1 × 10^8^) by intragastric gavage once every two days for a total of six injections. The treated mice were euthanized using a gradual fill method of CO_2_ exposure. The gastric tissues were prepared for hematoxylin-eosin (H&E) and immunohistochemistry (IHC) staining, as described previously [28]. Gastric tissue sections were prepared and stained with antibodies specific to IL-33 and ST-2 for 24 h at 4 °C, and then probed with a horseradish peroxidase-labeled goat anti-rabbit secondary antibody (Epitomics, Burlingame, CA, USA) and developed with an ABC kit (Vector Laboratories, Burlingame, CA, USA) [29]. 

### 2.8. Statistical Analysis

The experimental results are presented as mean ± standard deviation of independent triplicate experiments. The Student’s *t*-test was performed to calculate the statistical significance of differences between the two groups. A *p*-value of less than 0.05 was considered statistically significant. The statistical software was the SPSS program (version 12.0 for windows, SPSS Inc., Chicago, IL, USA).

## 3. Results

### 3.1. H. pylori Induces IL-33 Expression in Human Gastric Epithelial Cells

**Figure 1:**
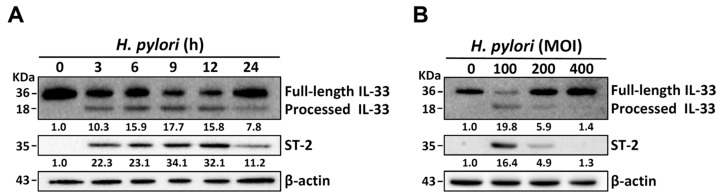
*H. pylori* induces Interleukin (IL)-33 and suppression of tumorigenicity-2 (ST-2) expression in gastric epithelial cells. AGS cells were infected with *H. pylori* (**A**) at a multiplication of infection (MOI) of 100 for the indicated times or (**B**) at different MOIs for 9 h. Total cell lysates were prepared to analyze the expression levels of IL-33 and ST-2 by Western blot. Molecular weights of full-length IL-33 and processed IL-33 were 36 KDa and 18 KDa, respectively. β-actin was used as an internal control. The expression levels of processed IL-33 and ST-2 were quantified by the signal intensity and indicated at the bottom of each lane.

We examined whether *H. pylori* induces the production of processed IL-33 in gastric epithelial cells using three human stomach-derived cell lines: AGS, SCM-1, and TSGH-9201. We found that *H. pylori*-induced production of processed IL-33 was significantly higher in AGS cells than in the other two cell lines (Figure S1). Therefore, AGS cells were selected as an assay platform for *H. pylori* infection in the following experiments. AGS cells were infected with *H. pylori* at an MOI of 100 for 0 to 24 h. Western blot showed a gradual increase in the expression levels of processed IL-33 and ST-2 following *H. pylori* infection in the first 9 h; however, upon infection for 12 to 24 h, the expression levels decreased (Figure 1A). We next examined whether *H. pylori* infection induced IL-33 and ST-2 expression. For this experiment, the cells were treated with *H. pylori* at MOIs of 0 to 400 for 9 h. As shown in Figure 1B, there was an increase in the expression of processed IL-33 and ST-2 in cells infected with *H. pylori* at an MOI of 100, but not at MOIs of 200 to 400. Furthermore, qRT-PCR showed that mRNA levels of IL-33 and ST-2 peaked at 9 h following *H. pylori* infection and reduced after incubation for 12 to 24 h (Figure S2). These results indicate that *H. pylori*-induced expression of processed IL-33 and ST-2 is time and MOI dependent. Therefore, the following conditions were adopted for all subsequent experiments: *H. pylori* (MOI = 100) infection for 9 h.

### 3.2. H. pylori Induces IL-33 Translocation from the Nucleus to the Cytoplasm

**Figure 2:**
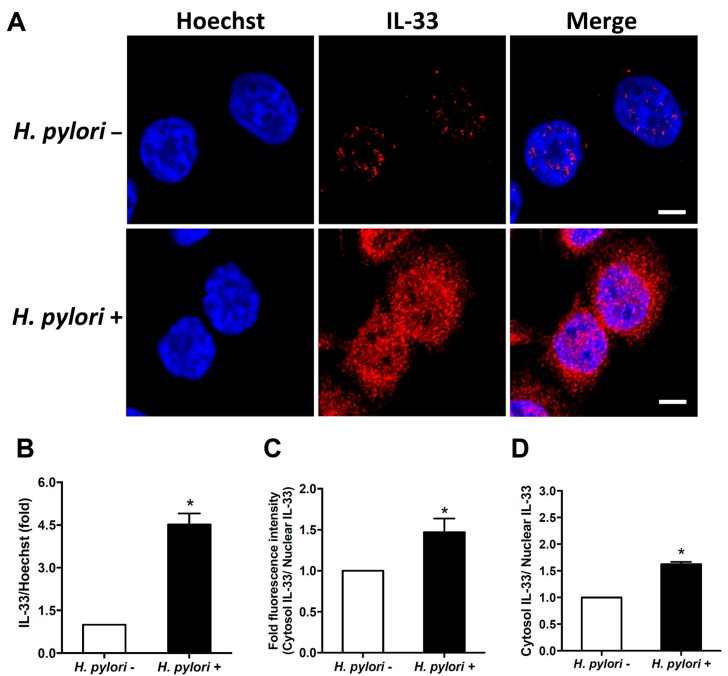
IL-33 translocates from the nucleus to cytoplasm in response to *H. pylori* infection. AGS cells were left untreated or infected with *H. pylori* at an MOI of 100 for 9 h. Cells were fixed and stained for IL-33 (red), then probed with Hoechst 33342 (blue) to identify the cell nucleus. (**A**) The stained cells were analyzed by confocal microscopy. Scale bar, 5 μm. (**B**) IL-33 (red) signal was quantified and normalized with Hoechst 33342. (**C**) The cytoplasmic IL-33 was quantified and normalized with nuclear fluorescence. Imaging data of arithmetic mean intensity were analyzed by using the ZEN-blue edition software (Carl Zeiss). (**D**) Cytoplasmic and nuclear fractions were analyzed to determine IL-33 levels by ELISA. *, *p* < 0.05.

It has been reported that IL-33 can translocate from the nucleus to cytoplasm [10]. We then explored IL-33 distribution in cells infected with *H. pylori* by confocal microscopy. A faint expression of IL-33 was observed in the nucleus of AGS cells before *H. pylori* infection (Figure 2A). Upon *H. pylori* infection, IL-33 was abundantly expressed in both the nucleus and cytoplasm (Figure 2B). Immunofluorescence assay and ELISA were performed to measure the cytoplasmic and nuclear IL-33 fractions. The results indicated that *H. pylori*-induced IL-33 translocation from the nucleus to the cytoplasm was significantly increased compared with uninfected cells (Figure 2C,D). These results indicate that *H. pylori* infection increases IL-33 level, which is highly expressed in the nucleus, and in turn, is translocated to the cytoplasm.

### 3.3. H. pylori Increases the Levels of IL-33 Receptor ST2 and Co-Receptor IL-1RAcP

**Figure 3:**
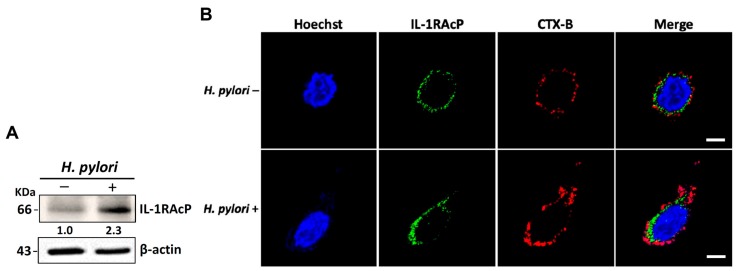
*H. pylori* induces IL-1 receptor accessory protein (IL-1RAcP) expression but not in lipid rafts. AGS cells were left untreated or infected with *H. pylori* at an MOI of 100 for 9 h. (**A**) Expression levels of IL-1RAcP and β-actin were assessed using Western blot. (**B**) Cells were stained for IL-1RAcP (green), Hoechst 33342 (blue), and cholera toxin subunit B (CTX-B) to label lipid rafts (red). The stained cells were analyzed by confocal microscopy. Scale bar, 5 μm.

**Figure 4:**
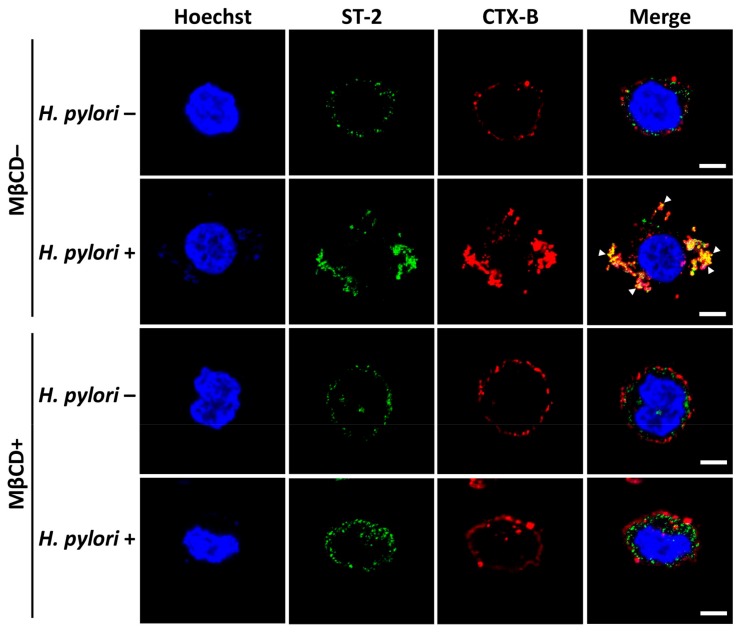
*H. pylori* elicits ST-2 mobilization into lipid rafts. AGS cells were left untreated or infected with *H. pylori* at an MOI of 100 for 9 h. Cells were subsequently stained for ST-2 (green), CTX-B (red), and Hoechst 33342 (blue). The stained cells were analyzed by confocal microscopy. The co-localization of ST-2 with CTX-B appears yellow in the merged image. Scale bar, 5 μm.

IL-33 receptors ST2 and IL-1RAcP are present on epithelial cells. Binding of IL-33 to the receptors activates NF-κB signaling [30]. We, therefore, analyzed whether *H. pylori* increased IL-1RAcP expression in membrane rafts. As shown in Figure 3A, *H. pylori* infection increased IL-1RAcP expression in AGS cells. However, confocal microscopy revealed that *H. pylori*-induced IL-1RAcP did not localize to the cholesterol-rich microdomains of the cell membrane (Figure 3B). In contrast, we observed that ST-2 mobilized into the membrane rafts in response to *H. pylori* infection (Figure 4). The depletion of cholesterol by MβCD led to a reduction in *H. pylori*-induced ST-2 expression in the membrane rafts (Figure 4).

**Figure 5:**
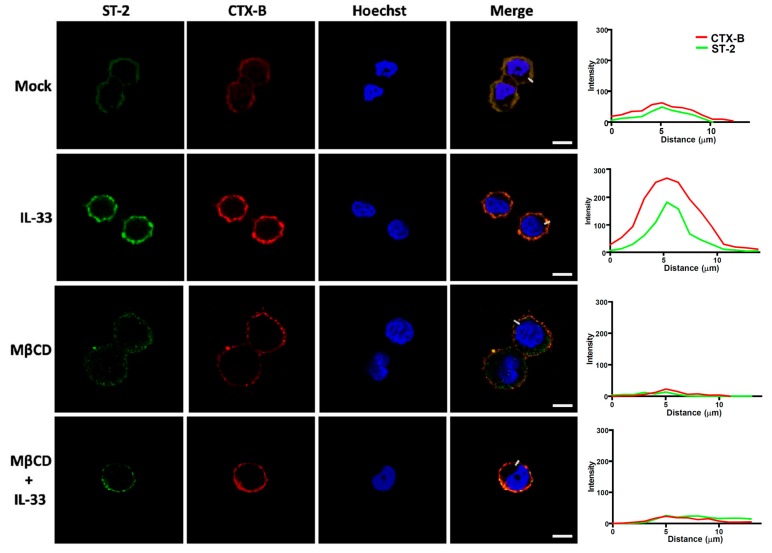
Recruitment of ST-2 into the lipid rafts is induced by IL-33. AGS cells were pretreated with or without 5 mM MβCD followed by incubation with recombinant IL-33 (100 ng/mL) at 11 °C for 1 h. Cells were then stained for ST-2 (green), CTX-B (red), and Hoechst 33342 (blue). Fluorescence distributions of ST-2 (green) and CTX-B (red) signals across the white lines were analyzed and exhibited as a line intensity histogram in the right panel. Scale bar, 10 µm.

We then examined whether IL-33 itself could prompt ST-2 co-localization to the membrane rafts. AGS cells were incubated with 100 ng/ml of recombinant IL-33 at 11 °C for 1 h to maintain membrane fluidity and prevent internalization [16]. IL-33 increased expression of ST-2, which was colocalized with CTX-B on the membrane (Figure 5). IL-33-induced ST-2 expression was reduced in cells pretreated with MβCD. Together, these results indicate that *H. pylori* infection increases IL-33 production and recruits ST-2, but not IL-1RAcP, into the membrane rafts.

### 3.4. Sufficient Cholesterol is Crucial for H. pylori-Induced IL-8 and IL-33 Production

**Figure 6:**
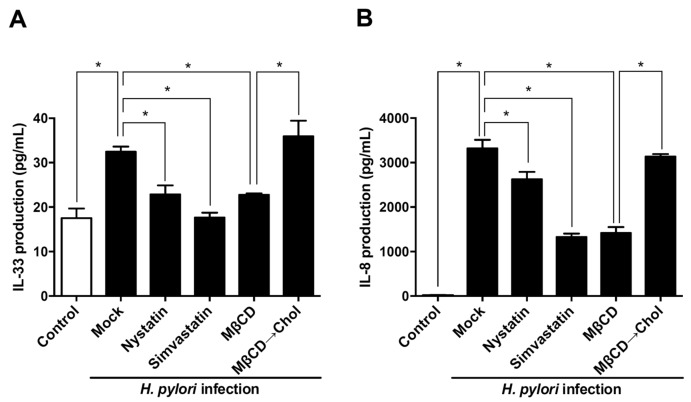
Sufficient cholesterol in the membrane rafts is crucial for *H. pylori*-induced IL-33 and IL-8 production. AGS cells were left untreated or pretreated with nystatin (50 μg/mL), simvastatin (10 μM), or MβCD (5.0 mM), or treated MβCD followed by replenishment of water-soluble cholesterol (400 μg/mL). After *H. pylori* infection at an MOI of 100 for 9 h, the expression levels of (**A**) IL-8 and (**B**) IL-33 were determined by ELISA. *, *P* < 0.05.

We further explored whether membrane rafts were required for *H. pylori*-induced IL-33 and IL-8 production. As shown in Figure 6, disruption of lipid rafts by nystatin, simvastatin, and MβCD significantly reduced IL-8 production in *H. pylori*-infected cells. Replenishing normal cholesterol levels reversed the inhibitory effect of methyl-β-cyclodextrin (MβCD) on IL-8 secretion. A similar effect was observed for IL-33. Our results indicate that the presence of sufficient cholesterol in membrane rafts is crucial for *H. pylori*-induced IL-8 and IL-33 production.

### 3.5. H. pylori Increases the Level of IL-33 Receptor ST2 and Co-Receptor IL-1RAcP

**Figure 7:**
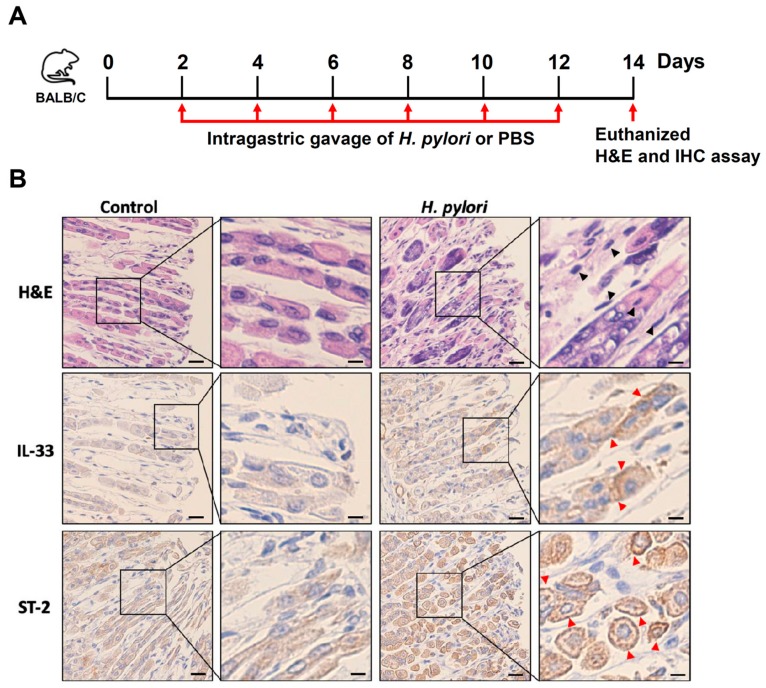
*H. pylori* infection induces IL-33 and ST-2 expression in mouse gastric epithelial cells. (**A**) Mice were infected with *H. pylori* (1 × 10^8^) by intragastric gavage once every 2 days for a total of six administrations. (**B**) Tissue sections of the stomach were fixed and stained with H&E, or prepared for IHC staining with specific antibodies against IL-33 and ST-2. The magnified images are shown in the right panel of each cropped image. Inflammatory cell infiltration in the gastric epithelium was observed (black arrowheads), along with evidence of both IL-33 and ST-2 expression in the gastric tissues (red arrowheads). Scale bars in left panels, 20 μm, and in magnified right panels, 60 μm.

To understand if the effects of *H. pylori*-induced IL-33 expression can be replicated in vivo and induce stomach inflammation, we infected mice with *H. pylori* (1 × 10^8^) by intragastric gavage once every 2 days for a total of six administrations (Figure 7A). Mice were euthanized on day 14, and the gastric tissues were histologically analyzed. H&E staining showed no inflammatory leukocytes in the gastric epithelium of the control mice (Figure 7B). In contrast, a severe inflammatory cell infiltration was observed in the gastric tissues of *H. pylori*-infected mice. IHC staining revealed a significant increase in IL-33 and ST-2 levels in the gastric tissue of *H. pylori*-infected mice compared to the control group. In conclusion, we demonstrated that *H. pylori*-induced IL-33 increased the level of ST-2 in the membrane rafts, and this event was crucial for inducing inflammation in the stomach.

## 4. Discussion

Although IL-33 plays a pivotal role in *H. pylori* infection in mast cells and gastric epithelial cells [10,11], the involvement of membrane rafts in *H. pylori*-induced IL-33/ST-2 signaling and its effect on inflammation had not been investigated before this study. To the best of our knowledge, this study demonstrates for the first time that sufficient cholesterol level is essential for *H. pylori*-mediated activation of IL-33/ST-2-induced inflammation. Because targeting the IL-33/ST-2 axis has been proposed for the treatment of several diseases [31,32], understanding the exact role of IL-33/ST-2 in *H. pylori*-induced pathogenesis is particularly important. 

IL-33 acts as an endogenous danger signal that is released after cell damage to alarm and activate the immune system during microbial infections [4]. IL-33 binding to its receptor ST-2 and co-receptor IL-1RAcP leads to the activation of the NF-κB signaling pathway and subsequent immune cell activation [5]. IL-33 can be released by cells infected with certain pathogens, including *Pseudomonas aeruginosa*, *Staphylococcus aureus*, *Leptospira interrogans,* and *Cryptococcus neoformans* [33,34,35,36]. In agreement with previous studies, we demonstrated that IL-33/ST-2/IL-1RAcP expression was upregulated in *H. pylori*-infected human gastric epithelial cells. Elevated levels of IL-33 suppressed microbial colonization and ameliorated pathogenesis in different animal models [34,37]. Moreover, increased IL-33 levels suppressed immune responses and decreased mortality in a mouse model of experimental sepsis [38]. Collectively, these results indicate that IL-33 plays a crucial role during bacterial infection of cells and that it also contributes to immune defense against pathogens.

IL-33 is predominantly a nuclear factor with transcriptional regulatory properties [39]. Our results showed that IL-33 can be translocated from the nucleus to the cytoplasm and secreted by gastric epithelial cells following *H. pylori* infection. The levels of processed IL-33 and ST-2 in these cells peaked at 9 h following *H. pylori* infection. However, after 12 h, IL-33 and ST-2 levels reduced gradually. A previous study reported that *H. pylori* infection caused the loss of nuclear IL-33 and promoted the release of processed IL-33 [10]. Although processed IL-33 can be secreted extracellularly, the detailed mechanisms through which proteases cleave IL-33 in gastric epithelial cells require further investigation. 

The physiological role of IL-33 has been reported in animal models of *H. pylori* infection. However, results so far have been contradictory. IL-33 is a potent inducer of proinflammatory cytokines released in mast cells [40,41]. The receptors of IL-33 include ST-2 and IL-1RAcP, which are abundantly expressed in mast cells and Th2 cells [5]. IL-33 administration to mast cells activated the NF-κB and MAP signaling pathways, leading to the initiation of inflammation and the progression of several diseases, such as asthma, allergy, anaphylaxis, and microbial infections [30]. Moreover, mast cells were involved in *H. pylori*-induced gastrointestinal diseases [42]. Most importantly, a recent study demonstrated that *H. pylori*-induced IL-33 promoted TNF-α secretion from mast cells and that this facilitated bacterial colonization and inflammation in the stomach [11]. Given the crucial functions of mast cells, these findings suggest that IL-33 plays a central role in gastric *H. pylori* infection. Our results were in agreement with these reports that IL-33 production was increased following *H. pylori* infection and contributed to the inflammatory response. 

Although IL-33 reportedly plays a pivotal role in gastritis exacerbation, a recent study revealed that NOD1 is required for *H. pylori*-induced IL-33 production and that this confers a protective role against inflammation [10]. This finding was supported by other studies showing that IL-33 could promote M2 macrophage polarization and Treg proliferation, both of which are involved in the suppression of the inflammation [43,44]. These contradictory roles of IL-33 in the regulation of immune response against *H. pylori* infection can be explained by the fact that IL-33 induces the activation of group 2 innate lymphoid cells (ILC2) and Th2 cytokine response in acute *H. pylori* infection. However, a Th1-skewed response occurs instead in chronic *H. pylori* infection [45,46]. IL-33 is a crucial regulator of the immune and inflammatory responses and serves diverse functions that orchestrate deteriorative or protective effects [6]. Thus, the precise role of IL-33 in either the acute or chronic *H. pylori* infection warrants further investigation.

Cholesterol usurpers or disruptors interfere with the lipid raft constituents and can decrease the risk of bacterial infections [47,48,49]. Based on a recent nationwide case-control study by us, patients who were prescribed with statins (inhibitors against HMG-CoA reductase for lowering cholesterol) exhibited a significantly reduced risk of *H. pylori*-associated peptic ulcer diseases and gastric cancers [20,22]. The current study subsequently showed that there was a reduction in the expression of IL-33/ST2 elicited by *H. pylori* upon statin treatment. Statins promote autophagy fusion with lysosomes to reduce bacterial burdens in macrophages [26,50]. IL-33 treatment enhances autophagy in mice with experimental colitis [51]. Intranasal administration of IL-33 was shown to promote IL-13-dependent autophagy and that this axis regulated mucus secretion by airway epithelial cells [52]. Because autophagy is closely intertwined with immune regulation, cytokines may be implicated in this interaction [53]. However, whether lipid rafts involved in *H. pylori*-induced IL-33 expression can regulate autophagy and initiate an inflammatory response in gastric epithelial cells remains unclear. The role of autophagy in how *H. pylori* exploits lipid rafts to trigger IL-33 production was beyond the scope of this study, but it deserves further investigation in the future.

## 5. Conclusions

This study demonstrated that *H. pylori* infection increased IL-33/ST-2 expression in gastric epithelial cells. IL-33 expression was localized in both the cytoplasm and nucleus, whereas ST-2 was recruited to the membrane rafts. Most importantly, in vivo studies revealed increased IL-33/ST-2 expression and leukocyte infiltration in *H. pylori*-infected mice. Together, these results demonstrate that ST-2 is mobilized into lipid rafts in response to *H. pylori*-induced IL-33 production, exacerbating inflammation in the stomach. Unveiling the mechanism of *H. pylori*-host interactions may pave an avenue for developing novel therapeutic modalities to control *H. pylori* infection.

## Supplementary Materials

The following are available online at https://www.mdpi.com/2073-4409/8/10/1290/s1, Figure S1: *H. pylori* induces IL-33 expression in gastric epithelial cells, Figure S2: *H. pylori* induces IL-33 and mST-2 mRNA expression in gastric epithelial cells.
