# Supplementary material for: Helicobacter pylori Induces IL-33 Production and Recruits ST-2 to Lipid Rafts to Exacerbate Inflammation
Source: Cells. 2019 Oct 21;8(10):1290. doi: 10.3390/cells8101290 (PMC6830106; doi:10.3390/cells8101290)
Supplement: Supplementary file 1 [file cells-08-01290-s001.pdf]

Helicobacter pylori Induces IL-33 Production and Recruits ST-2 to Lipid Rafts to Exacerbate Inflammation

Chia-Jung Kuo, Chun-Ya Chen, Horng-Ren Lo, Chun-Lung Feng, Hui-Yu Wu, Mei-Zi Huang, Tung-Nan Liao, Yu-An Chen, Chih-Ho Lai

Supplementary Materials

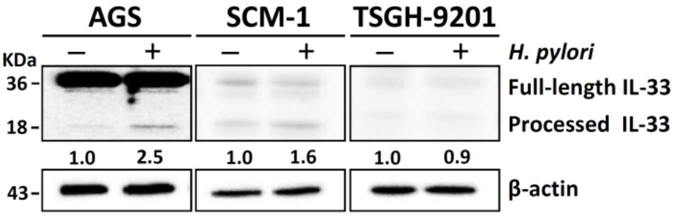

Figure S1. *H. pylori* induces IL-33 expression in gastric epithelial cells. Three gastric epithelial cell lines, including AGS, SCM-1, and TSGH9201 cells were respectively infected with *H. pylori* at an MOI of 100 for 9 h. The protein expression levels of full-length and processed IL-33 were determined by western blot. β-actin was used as an internal control. The expression levels of processed IL-33 were quantified by the signal intensity and indicated at the bottom of each lane.

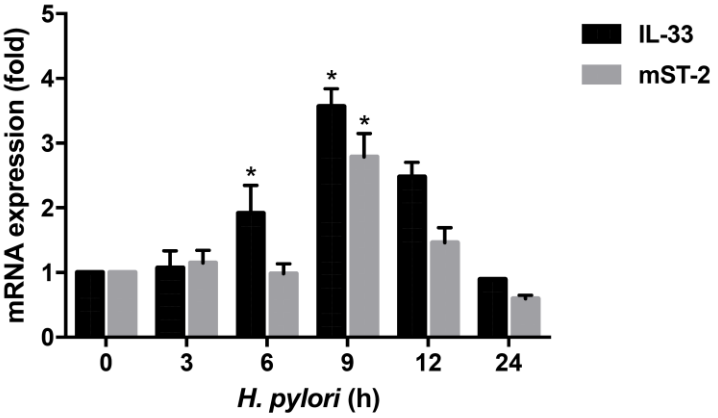

Figure S2. *H. pylori* induces IL-33 and mST-2 mRNA expression in gastric epithelial cells. AGS cells were infected with *H. pylori* for the indicated times (0–24 h). mRNA levels of IL-33 and mST-2 were analyzed by using quantitative real-time PCR. Results were expressed as mean ± standard deviations. *, $P < 0.05$ as compared to each *H. pylori* uninfected group.
